# The Knowledge, Attitude, and Practice Regarding Deep Vein Thrombosis Among Pregnant and Postpartum Women: The Mediating Effect of Attitude

**DOI:** 10.1002/nop2.70351

**Published:** 2025-11-14

**Authors:** Mei Xu, Longfeng Fang, Yaguai Wu, Taoling Yan

**Affiliations:** ^1^ The Second Affiliated Hospital of Xiamen Medical College Xiamen China

**Keywords:** cross‐sectional study, health knowledge, attitudes, practice, postpartum period, pregnancy, thrombosis

## Abstract

**Aim:**

To assess the knowledge, attitude, and practice (KAP) regarding deep vein thrombosis (DVT) among pregnant and postpartum women in Xiamen, China.

**Design:**

A cross‐sectional study using self‐designed questionnaires was conducted.

**Methods:**

From October 18 to October 28, 2023, questionnaires were distributed to pregnant and postpartum women. Logistic regression and structural equation modelling (SEM) were used to analyse factors associated with KAP scores.

**Results:**

A total of 609 participants completed the study. The average scores were: knowledge 4.19/11, attitude 36.38/50, and practice 26.68/35. Higher knowledge, higher education, and higher income were independently associated with higher practice scores. The SEM showed that knowledge directly positively affected attitude and practice, and influenced practice indirectly through attitude.

**Conclusions:**

Pregnant and postpartum women in Xiamen have poor knowledge but neutral attitudes and acceptable practices regarding DVT. Knowledge is linked to attitude and practice, with attitude mediating the effect of knowledge on practice. Awareness campaigns to improve attitudes are essential for promoting preventive practices.

**Reporting Method:**

We have adhered to relevant EQUATOR guidelines and STROBE reporting methods.

**Patient or Public Contribution:**

No patient or public contribution.

## 1 Introduction

Venous thromboembolism, commonly manifesting as deep vein thrombosis (DVT) and pulmonary embolism (PE), affects up to 5% of the population worldwide and nearly 10 million people every year (Khan et al. 2021). Among other factors, pregnancy and childbirth increase the relative risk of DVT, while postpartum period is characterised by the highest daily incidence (Gris et al. 2022; Kalaitzopoulos et al. 2022). With an estimated incidence of approximately 1–2 per 1000 pregnancies, the above risk is reportedly almost 5 times higher in pregnant women compared to non‐pregnant (Alsheef et al. 2022; Kilkenny and Frishman 2024), accounts for up to 10% of maternal mortality in industrialised countries (Gris et al. 2022).

Anticoagulation therapy is usually prescribed to control DVT symptoms and reduce the risk of complications (Linnemann et al. 2024; Stevens et al. 2024); in patients with a long‐term risk of recurrent DVT, such as those with active cancer or potent thrombophilia, indefinite anticoagulant treatment might be advised (Khan et al. 2021; Rodger and Le Gal 2018). Despite the necessity of thromboprophylaxis for many women with a history of DVT, especially in subsequent pregnancies (Middleton et al. 2021), awareness, and understanding of venous thromboembolism risks, symptoms, and treatment options vary significantly among pregnant patients (Elgendy et al. 2020; Kevane and Áinle 2023). This variation may influence their engagement with preventive measures, symptom reporting, and adherence to prescribed treatments (Choy et al. 2022). In addition, excluding pregnant women from clinical trials on anticoagulants has further increased the knowledge gaps (Edebiri and Ní Áinle 2022). Improving patient education on DVT during pregnancy is critical to fostering proactive healthcare behaviours and supporting early intervention strategies.

As almost all current guidelines are based on scarce evidence and vary in their approach (Middleton et al. 2021; Wiegers and Middeldorp 2020), it is essential to ensure the cooperation and exchange of knowledge between doctors and patients in the treatment and prevention of DVT in pregnant and postpartum women. Knowledge, attitude, and practice (KAP) study is a structured approach that may provide important information on knowledge, beliefs and opinions, behaviours and experiences, or personal attributes of patients (or medical personnel) on a certain condition (Andrade et al. 2020; Santesso et al. 2020). A few previous KAP studies already addressed the issue of DVT in pregnancy, both among healthcare providers and patients. In particular, Al‐Mugheed and Bayraktar (2018) reported inadequate knowledge of DVT risks and poor practices of DVT prevention in nurses, highlighting the urgent need for educational measures. Despite that, more recent studies (Kim and Kim 2019; Ojukwu et al. 2023; Ramadan et al. 2019) still report the notable lack of awareness, as well as mixed or negative attitudes, most likely related to the existing knowledge gaps surrounding DVT prevention, suggesting that educational interventions alone may be neither sufficient nor optimally effective in improving the practice of DVT prevention among pregnant and postpartum women. According to the theory of planned behaviour (Bosnjak et al. 2020), attitude may play a role as a critical mediating factor in bridging the gap between knowledge and practice, underscoring the need for innovative educational strategies that go beyond simply disseminating information, focusing instead on shaping positive attitudes and fostering behavioural changes. Integrating attitudinal and motivational components into patient education delivered by nurses and midwives may enhance their impact (Sandall et al. 2024), enabling these providers to address both informational gaps and the underlying beliefs that influence DVT prevention practices among pregnant and postpartum women. Given the scarcity of research specific to the Chinese population, additional studies would provide essential, context‐specific data to guide culturally tailored education and clinical guidelines. Such evidence would allow nursing and midwifery teams to design and implement more effective, population‐relevant interventions, ultimately improving maternal care and reducing thrombotic complications.

Therefore, this cross‐sectional study aimed to investigate knowledge, attitude, and practice on DVT among pregnant and postpartum women in Xiamen, China. The specific objectives were to: (1) Assess the level of knowledge, attitude, and practice regarding DVT in this population; (2) Identify sociodemographic factors associated with variations in KAP scores; (3) Explore the interrelationships between knowledge, attitude, and practice using structural equation modelling, with a particular focus on the mediating role of attitude.

## 2 Materials and Methods

### 2.1 Study Design and Participants

This cross‐sectional study was conducted in Xiamen, China between October 18, 2023, and October 28, 2023. The questionnaires were distributed among women who were consulted during pregnancy and the postpartum period. The inclusion criteria were: (1) Pregnant women or postpartum within 3 months after delivery; (2) Volunteered to participate in this study. Questionnaires with incomplete answers or those filled out by participants who were unable to understand the questions were excluded.

The present study received the ethical approval of the Medical Ethics Committee of the author's hospital. All participants signed the written informed consent form before participating.

### 2.2 Questionnaire

The questionnaire was designed with reference to the previous study by Kingman and Economides (2003) on pregnant women's experiences and knowledge of health issues, and the study by Mellon et al. (2020) on awareness of pregnancy‐associated health risks among pregnant women, as well as the current Chinese guidelines on DVT. Before the start of the study, 47 questionnaires were administered to randomly selected participants for reliability testing, with a Cronbach's α of 0.797, suggesting good reliability (Taber 2018).

The final questionnaire, in Chinese, comprised four following dimensions: demographic characteristics (age, history of pregnancy, height, weight, residence, ethnicity, education, occupation, monthly income, medical insurance, underlying diseases, smoking, and history of surgery), knowledge dimension, attitude dimension, and practice dimension. The knowledge dimension included 12 questions, where questions 1–11 were awarded 1 point for a correct answer and 0 points for an incorrect answer. Question 12 was used to evaluate the validity of the questionnaire. The total score ranged from 0 to 11 points. The attitude dimension contained 10 questions that were evaluated using the Likert five‐point scale. Among them, Question 1, Question 6, and Question 10 were positive, where 5 points indicated strongly agree and 1 point strongly disagree. The other questions were negative. The total score ranged from 10 to 50 points. The practice dimension consisted of 7 questions that were evaluated using the Likert five‐point scale, from always (very willing) to never (very unwilling). The total score ranged from 7 to 35 points. To assess the overall KAP of participants, the knowledge was defined as poor (≤ 60%), moderate (60%–80%), and good (≥ 80%); the attitude was defined as negative (≤ 60%), neutral (60%–80%), and positive (≥ 80%); and the practice was classified as inappropriate (≤ 60%), acceptable (60%–80%), and appropriate (≥ 80%) according to the modified Bloom's cutoff standards.

A convenience sampling method was employed to recruit participants. Paper‐based questionnaires were distributed to pregnant and postpartum women who were either hospitalised in the obstetrics and gynaecology department of the authors' hospital or who visited the hospital for antenatal or postnatal care during the study period. Participation was voluntary for those able to understand and complete the questionnaire in Chinese.

### 2.3 Sample Size Calculation

The sample size was calculated based on item‐to‐respondent theory, with ratios between 1:5 and 1:20 considered suitable (Naqvi et al. 2020). Thus, a ratio of 1:20 was selected and with 28 KAP items of the questionnaire (excluding demographics and the question used to evaluate the validity), the required sample size was 560. The annual visit rate at the study site is ~4000 patients, and convenience sampling was used during the study period.

### 2.4 Statistical Analysis

SPSS 26.0 (IBM, Armonk, NY, USA) was used for statistical analysis. Continuous data were expressed as Mean ± SD, and compared by one‐way ANOVA. Categorical data were expressed as *n* (%). Logistic regression analysis was conducted to identify sociodemographic factors independently associated with higher practice scores, as practice was treated as a binary outcome (high vs. low) for ease of interpretation and clinical relevance. Variables significant on univariable analysis were entered into logistic regression. Structural equation modelling (SEM) was employed to assess the hypothesised relationships among knowledge, attitude, and practice, as it allows for simultaneous estimation of multiple dependent relationships. In this study, SEM enabled us to test whether attitude mediates the effect of knowledge on practice while accounting for measurement error and latent constructs and providing a more comprehensive understanding of the interrelationships among the KAP components. For all analyses two‐sided *p* < 0.05 was considered statistically significant.

## 3 Results

A total of 629 respondents participated in this study, and 20 questionnaires were excluded due to incomplete data or inconsistent answers, and the final analysis included 609 valid questionnaires. The majority of these women were of Chinese Han ethnicity (93.76%), never smoked (96.72%), and were younger than 30 years of age (62.23%). One‐third of participants (33.99%) had a history of previous surgery (Table 1).

**TABLE 1:** Demographic characteristics and KAP scores of participants.

	*N* (%)	Knowledge		Attitude		Practice	
		Mean ± SD	*p*	Mean ± SD	*p*	Mean ± SD	*p*
*Age*			0.615		0.144		0.748
≤ 30	379 (62.23)	4.14 ± 3.076		36.62 ± 5.203		26.72 ± 3.691	
> 30	230 (37.77)	4.27 ± 3.203		36.00 ± 4.790		26.62 ± 3.345	
*Pregnant for the first time*			0.071		0.150		0.133
Yes	265 (43.51)	4.45 ± 3.142		36.72 ± 5.175		26.93 ± 3.683	
No	344 (56.49)	3.99 ± 3.098		36.13 ± 4.954		26.49 ± 3.458	
*Ethnicity*			0.818		0.213		0.257
Chinese Han	571 (93.76)	4.20 ± 3.111		36.45 ± 5.045		26.64 ± 3.534	
National minority	38 (6.24)	4.08 ± 3.332		35.39 ± 5.186		27.32 ± 3.946	
*Occupation*			< 0.001		0.032		0.001
Office staff and related personnel	309 (50.74)	4.67 ± 3.229		36.82 ± 4.722		27.17 ± 3.442	
Others	300 (49.26)	3.70 ± 2.933		35.94 ± 5.349		26.18 ± 3.619	
*Residence*			0.090		0.846		0.143
Non‐urban	276 (45.32)	3.96 ± 3.153		36.34 ± 5.052		26.45 ± 3.717	
Urban	333 (54.68)	4.39 ± 3.089		36.42 ± 5.066		26.87 ± 3.421	
*Education*			< 0.001		< 0.001		< 0.001
Technical secondary school or below	302 (49.59)	3.47 ± 2.896		35.26 ± 4.981		25.64 ± 3.523	
College or above	307 (50.41)	4.91 ± 3.177		37.49 ± 4.892		27.70 ± 3.299	
*Monthly income, (Yuan)*			0.022		0.002		0.001
< 5000	170 (27.91)	3.60 ± 3.074		35.52 ± 5.134		25.92 ± 3.745	
5000–10,000	294 (48.28)	4.49 ± 3.183		36.29 ± 4.986		26.76 ± 3.479	
10,000–20,000	112 (18.39)	4.41 ± 2.970		37.82 ± 4.989		27.18 ± 3.197	
> 20,000	33 (5.42)	3.88 ± 3.008		36.79 ± 4.595		28.18 ± 3.795	
*Smoking*			0.051		0.005		0.673
Yes	589 (96.72)	4.24 ± 3.135		36.49 ± 5.011		26.69 ± 3.554	
No	20 (3.28)	2.85 ± 2.434		33.25 ± 5.505		26.35 ± 3.856	
*Medical insurance*							
National health insurance	564 (92.61)	4.29 ± 3.111	0.008	36.46 ± 5.048	0.206	26.81 ± 3.501	0.001
Commercial medical insurance	27 (4.43)	4.19 ± 3.340	0.991	36.81 ± 4.764	0.651	26.41 ± 3.296	0.683
None	39 (6.40)	2.82 ± 2.855	0.004	35.00 ± 5.282	0.077	24.92 ± 3.821	0.001
*History of surgery*			0.768		0.859		0.534
Yes	207 (33.99)	4.14 ± 3.183		36.33 ± 5.042		26.81 ± 3.482	
No	402 (66.01)	4.22 ± 3.095		36.41 ± 5.069		26.62 ± 3.604	

The average knowledge score was 4.19 ± 3.123 (total score: 11), the average attitude score was 36.38 ± 5.056 (total score: 50), and the average practice score was 26.68 ± 3.562 (total score: 35).

The highest correct rates in the knowledge dimension were only 65.19% and 63.05% for questions “Factors such as advanced age, obesity, surgical history, long‐term bed rest, and smoking will increase the risk of deep vein thrombosis” and “Deep vein thrombosis can be prevented by walking and increasing exercise”, respectively. The question with the lowest correct rate of 16.42% was “In vitro fertilization increases the risk of deep vein thrombosis” (Table 2).

**TABLE 2:** Answers from the knowledge dimension.

Knowledge	Correct *N* (%)
1. Leg pain, swelling, and discoloration are common symptoms of deep vein thrombosis	231 (37.93)
2. Different thicknesses of lower limbs and fatigue after walking are early manifestations of deep vein thrombosis	251 (41.22)
3. In‐vitro fertilisation increases the risk of deep vein thrombosis	100 (16.42)
4. Factors such as advanced age, obesity, surgical history, long‐term bed rest, and smoking will increase the risk of deep vein thrombosis	397 (65.19)
5. Severe vomiting during pregnancy increases the risk of deep vein thrombosis	124 (20.36)
6. Hypercoagulable state and venous stasis during pregnancy will increase the risk of deep vein thrombosis	299 (49.10)
7. Caesarean section may increase the risk of blood clots during the puerperal period	214 (35.14)
8. Deep vein thrombosis can be prevented by physical means (such as IPC, elastic stockings, etc.).	244 (40.07)
9. Deep vein thrombosis can be prevented by anticoagulant drugs such as warfarin during pregnancy	150 (24.63)
10. Deep vein thrombosis can be prevented by walking and increasing exercise	384 (63.05)
11. Low‐molecular‐weight heparin can be used for the prevention of deep vein thrombosis during pregnancy	159 (26.11)

In the attitude dimension, most participants agreed that DVT requires special attention and must be prevented during pregnancy and postpartum periods. Only a few participants agreed with the following statements: “People who have been bedridden for a long time will suffer from deep vein thrombosis, and I don't need to worry about it in particular” (5.42% agreed, 1.81% strongly agreed) and “All obese women will suffer from DVT” (7.88% agreed, 3.61% strongly agreed). Almost one‐third of participants (31.53%) agreed or strongly agreed that they don't want to use pharmacological treatment because it could affect their child (Table 3).

**TABLE 3:** Answers for the attitude dimension.

Attitude	Strongly agree	Agree	Neutrality	Disagree	Strongly disagree
1. Deep vein thrombosis can be fatal and requires special attention	176 (28.90)	271 (44.50)	143 (23.48)	13 (2.13)	6 (0.99)
2. Deep vein thrombosis generally occurs in older people, and I don't need to worry when I am young	14 (2.30)	32 (5.25)	132 (21.67)	345 (56.65)	86 (14.12)
3. All obese women will suffer from deep vein thrombosis	22 (3.61)	48 (7.88)	135 (22.17)	328 (53.86)	76 (12.48)
4. People who have been bedridden for a long time will suffer from deep vein thrombosis, and I don't need to worry about it in particular	11 (1.81)	33 (5.42)	139 (22.82)	338 (55.50)	88 (14.45)
5. The incidence of deep vein thrombosis is low, I don't need to worry	16 (2.63)	30 (4.93)	133 (21.84)	336 (55.17)	94 (15.44)
6. Pregnant women are a high‐risk group for deep vein thrombosis and need special attention	125 (20.53)	272 (44.66)	168 (27.59)	32 (5.25)	12 (1.97)
7. For pregnant women, physical means are enough to prevent deep vein thrombosis	25 (4.11)	59 (9.69)	222 (36.45)	264 (43.35)	39 (6.40)
8. I don't want to use drugs to prevent blood clots, as drugs have the potential to affect my child	35 (5.75)	192 (31.53)	234 (38.42)	123 (20.20)	25 (4.11)
9. During the period of childbirth I should rest in bed and move less	31 (5.09)	72 (11.82)	148 (24.30)	295 (48.44)	63 (10.34)
10. Adequate cooperation with medical staff can help prevent deep vein thrombosis	181 (29.72)	301 (49.43)	98 (16.09)	22 (3.61)	7 (1.15)

In the practice dimension, the majority of participants were still willing to receive pharmacological treatment for DVT if prescribed by a doctor (90.64%) and to increase exercising (86.20%). Avoiding high‐fat meals and staying in bed for a shorter time were less popular practices but were still practiced by the majority of respondents (Table 4).

**TABLE 4:** Answers for the practice dimension.

Practice	Always	Often	Sometimes	Occasionally	Never
1. I drink a lot of water on a daily basis	75 (12.32)	205 (33.66)	286 (46.96)	39 (6.40)	4 (0.66)
2. I try to wear loose clothes	175 (28.74)	317 (52.05)	103 (16.91)	12 (1.97)	2 (0.33)
3. I try to avoid staying in bed for a long time	76 (12.48)	229 (37.60)	224 (36.78)	70 (11.49)	10 (1.64)
4. I try to avoid high‐fat meals	81 (13.30)	210 (34.48)	232 (38.10)	70 (11.49)	16 (2.63)

To prevent deep vein thrombosis	Strongly agree	Agree	Neutrality	Disagree	Strongly disagree
5. I am willing to follow my doctor's instructions to take drugs	223 (36.62)	329 (54.02)	48 (7.88)	7 (1.15)	2 (0.33)
6. I am willing to accept physical interventions such as IPC, elastic stockings, etc.	131 (21.51)	270 (44.33)	174 (28.57)	29 (4.76)	5 (0.82)
7. I am willing to exercise more	172 (28.24)	353 (57.96)	71 (11.66)	12 (1.97)	1 (0.16)

The KAP patterns did not differ according to age, residence, or the number of previous pregnancies (all *p* > 0.05). However, the results showed significant differences in attitude and practice dimensions among participants with different education and income levels (Table 5). The multivariate analysis showed that higher education (OR = 2.341, 95% CI: [1.558–3.518], *p* < 0.001) was independently associated with higher knowledge scores; higher knowledge scores (OR = 1.137, 95% CI: [1.073–1.205], *p* < 0.001) and education (OR = 1.671, 95% CI: [1.147–2.434], *p* = 0.007) were independently associated with higher attitude scores; higher knowledge scores (OR = 1.125, 95% CI: [1.062–1.191], *p* < 0.001), education (OR = 1.880, 95% CI: [1.266–2.792], *p* = 0.002) and higher income (OR = 2.697, 95% CI: [1.188–6.123], *p* = 0.018) were independently associated with higher practice scores.

**TABLE 5:** Multivariate logistic regression analysis for knowledge, attitude, and practice.

Characteristics		Multivariate logistic regression	
		OR (95% CI)	*p*
*Knowledge*			
	*First pregnancy*		
	Yes	1.157 (0.811–1.652)	0.421
	No	Ref	
	*Occupation*		
	Office staff and related personnel	Ref	
	Others	0.851 (0.578–1.254)	0.415
	*Education*		
	Technical secondary school and below	Ref	
	Technical secondary school and above	2.341 (1.558–3.518)	< 0.001
	*Monthly income (Yuan)*		
	< 5000	Ref	
	5000–10,000	1.433 (0.929–2.211)	0.104
	10,000–20,000	1.017 (0.587–1.762)	0.953
	> 20,000	0.815 (0.349–1.902)	0.635
*Attitude*			
	*Knowledge scores*	1.137 (1.073–1.205)	< 0.001
	*Education*		
	Technical secondary school or below	Ref	
	College or above	1.671 (1.147–2.434)	0.007
	*Monthly income (Yuan)*		
	< 5000	Ref	
	5000–10,000	0.961 (0.618–1.493)	0.859
	10,000–20,000	1.558 (0.914–2.656)	0.103
	> 20,000	0.907 (0.387–2.127)	0.823
*Practice*			
	*Knowledge scores*	1.125 (1.062–1.191)	< 0.001
	*Attitude scores*	1.013 (0.978–1.050)	0.494
	*Occupation*		
	Office staff and related personnel	Ref	
	Others	0.986 (0.674–1.443)	0.942
	*Residence*		
	Non‐urban	Ref	
	Urban	1.138 (0.800–1.618)	0.473
	*Education*		
	Technical secondary school or below	Ref	
	College or above	1.880 (1.266–2.792)	0.002
	*Monthly income (Yuan)*		
	< 5000	Ref	
	5000–10,000	1.086 (0.712–1.658)	0.701
	10,000–20,000	1.302 (0.766–2.213)	0.330
	> 20,000	2.697 (1.188–6.123)	0.018

**FIGURE 1:**
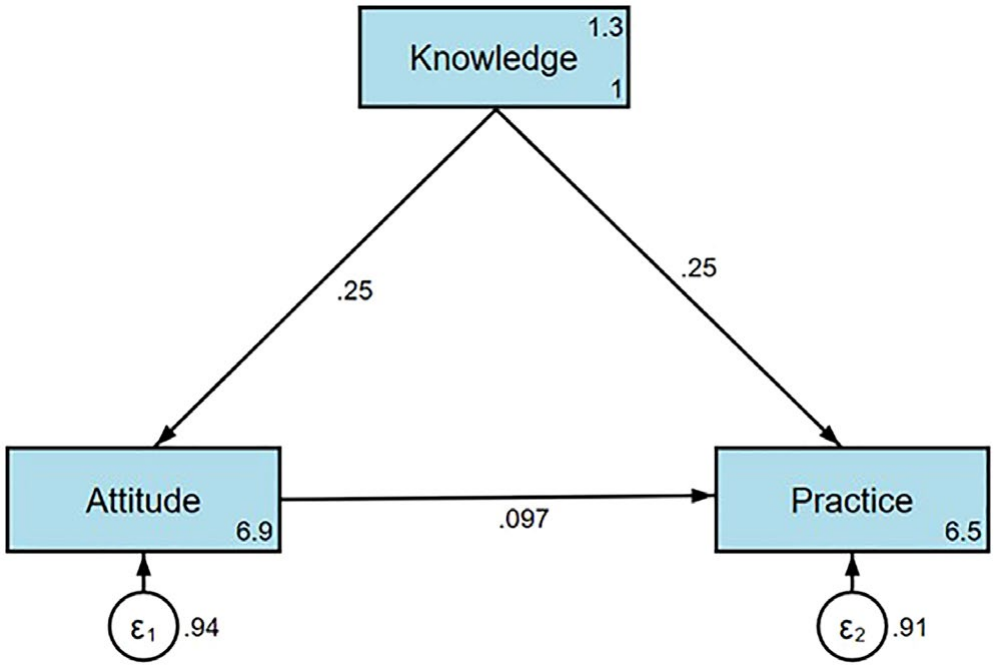
Structural equation model demonstrating direct and indirect effects of knowledge on practice, with the mediating role of attitude.

Application of SEM showed that knowledge had a direct positive effect on attitude (*β* = 0.249, 95% CI: [0.176–0.322], *p* < 0.001) and practice (*β* = 0.254, 95% CI: [0.179, 0.329], *p* < 0.001), while attitude had an additional effect on practice (*β* = 0.097, 95% CI: [0.019, 0.175], *p* = 0.015) (Table 6). The analysis (Figure 1) confirmed that knowledge also influenced practice indirectly through attitude (*β* = 0.024, 95% CI: [0.003, 0.045], *p* = 0.023).

**TABLE 6:** Structural equation model for mediation effects of knowledge on practice.

Model paths		Total effects		Direct effect		Indirect effect	
		*β* (95% CI)	*p*	*β* (95% CI)	*p*	*β* (95% CI)	*p*
*Attitude*							
	Knowledge	0.249 (0.176, 0.322)	< 0.001	0.249 (0.176, 0.322)	< 0.001		
*Practice*							
	Knowledge	0.278 (0.207, 0.350)	< 0.001	0.254 (0.179, 0.329)	< 0.001	0.024 (0.003, 0.045)	0.023
	Attitude	0.097 (0.019, 0.175)	0.015	0.097 (0.019, 0.175)	0.015		

## 4 Discussion

The present study showed that participants had poor knowledge but neutral attitudes and acceptable practice on DVT. In addition, areas for improvement, barriers, and demographic factors associated with lower or higher scores were discussed. To the best of our knowledge, this is the first KAP study on DVT in pregnancy or postpartum period that applied SEM to demonstrate the direct and indirect influence of knowledge on practice via attitude, confirming the mediating effect of attitude, and efficacy of specialised education. Findings contribute to the risk assessment of thrombotic burden, thus allowing consecutive advocacy on better education in DVT among the most vulnerable pregnant and postpartum women. By integrating these insights into patient education, nursing and midwifery‐led teams can more effectively teach pregnant and postpartum women about DVT risk, prevention strategies, and the importance of adherence. This proactive educational approach, embedded in everyday clinical encounters, can help reduce preventable thrombotic complications and improve maternal safety.

In this study the knowledge score of participants was only 38.01% from maximal, corresponding to the poor knowledge of pregnant and postpartum women regarding DVT prophylaxis—in line with recent reports in the comparable populations (Kim and Kim 2019; Ojukwu et al. 2023). Although thromboprophylaxis in pregnancy is actively researched, due to widely varying guidelines and approaches patients may be under‐ or over‐anticoagulated, eventually leading to poor outcomes (O'Keefe et al. 2022). Due to the weak evidence and lack of comprehensive clinical studies there is still no consensus on the diagnostic management of DVT during pregnancy (van der Pol et al. 2019). Moreover, some approaches—low‐molecular‐weight heparin specifically—also have questionable cost‐efficacy in China (Sun et al. 2021). Still, despite discouraging results, most participants in the present study were willing to receive pharmacological treatment if prescribed by a trusted doctor despite concerns regarding its potential to affect the child. These findings highlight the importance of educational interventions aimed at fostering meaningful communication with pregnant and post‐partum women about the necessity and benefits of thromboprophylaxis. Nurses and midwives, who often have the closest and most sustained contact with patients during antenatal and postnatal periods, are ideally positioned to translate clinical guidelines into understandable, actionable steps (Sandall et al. 2024). The KAP patterns in our study point to specific opportunities for nursing‐ and midwifery‐led education: structured teaching sessions to address the knowledge gap, counselling to reinforce motivation and shift attitudes toward stronger prevention behaviours, and practical demonstrations with follow‐up to improve consistency in practice. Such interventions should not only provide knowledge but also actively work to shape positive patient attitudes, which in the present study played a pivotal mediating role in translating understanding into effective adherence to practices. Recently developed risk assessment methods and scores (Gris et al. 2022) could be used more systematically to implement the above into decision‐making during pregnancy planning.

The answers given by the majority of participants in this study at least partly reflect knowledge gaps surrounding DVT prophylaxis in pregnancy. In particular, less than half of the participants (49.10%) correctly answered the knowledge question regarding “hypercoagulable state and venous stasis during pregnancy increasing the risk of DVT”, only a few of them disagreed (5.25%) or strongly disagreed (1.97%) with the attitude statement that “pregnant women are a high‐risk group for DVT and need special attention in terms of DVT prevention”. Secondly, the efficacy and safety of low‐molecular‐weight heparin in postpartum and breastfeeding periods are actively discussed (Gris et al. 2022; Wiegers and Middeldorp 2020). However, the question about low‐molecular‐weight heparin was answered correctly by only 26.11% of respondents in the present study, suggesting that recent advances in DVT treatment and prevention are not widely known in this clinical setting. Finally, it is important to note that although “in‐vitro fertilization increases the risk of deep vein thrombosis” (Grandone et al. 2025), that was mostly unknown to our participants (rate of correct answers only 16.42%). New educational interventions should consider the above, as IVF has become increasingly popular in China (Huang 2022).

In the previous report by Elgendy et al. (2020), pregnant and postpartum women with venous thromboembolism were less likely to present other risk factors, confirming that pregnancy itself was a major contributive factor. However, some acquired and inherited factors, such as autoimmune and inflammatory disorders, increased body mass index, prolonged immobilisation, miscarriages, and surgical intervention still contribute to DVT development in pregnancy (Cernera et al. 2020; Gris et al. 2022). In addition, our results showed significant differences in attitude and practice dimensions among participants with different education and income levels, suggesting that knowledge exchange with doctors is not the only factor influencing decision‐making regarding antithrombotic management. Socio‐economic factors may play a critical role in shaping health‐related attitudes and practices, with higher education and income potentially fostering more positive health behaviours (Wilderink et al. 2022). These findings underscore the importance of targeted educational interventions that address gaps in knowledge and encourage positive attitudes, especially among lower‐income or less‐educated groups, to ensure equitable health outcomes across diverse populations. Additional application of SEM in this study further confirmed direct and indirect influence of knowledge on practice via attitude, highlighting the mediating effect of attitude and suggesting that specialised education might be effective in increasing understanding of DVT prophylaxis among pregnant and postpartum women.

The present study has some limitations. Firstly, this was a single‐center study, and although the sample was relatively big, the specific population might have influenced the credibility and applicability of the results. As convenience sampling was used, selection bias may be present, and the findings may not be generalizable to the broader population. Secondly, study design allows for social expectation bias, as questions might have been answered from the point of the “better” practice. Accordingly, credibility depends on the likelihood that it provides an accurate picture of self‐reported practices (Santesso et al. 2020). Finally, even though investigators used balanced strategies to develop questions, the instrument was self‐developed and not standardised, so there is still a risk that some important factor was overlooked. Therefore, before generalising these findings, decision‐makers should carefully assess the degree of similarity between the study population and the target population for any intended application. Finally, due to the cross‐sectional design of the study, causal relationships cannot be inferred from the identified associations.

## 5 Conclusions

In conclusion, pregnant and postpartum women included in this study had poor knowledge of DVT but neutral attitudes and acceptable practices, while knowledge was independently linked to both attitude and practice, with attitude serving as a critical mediator influencing practical actions. Although the vast majority of participants believed that pharmacological prevention might harm the fetus, they were willing to receive treatment if prescribed by a trusted doctor; interventions should be designed not only to increase knowledge but also to positively influence attitudes, a key mediator between knowledge and practice. Relevant topics that should be discussed during future interventions were identified, including specific risk factors, necessity and impact of thromboprophylaxis, as well as the increased DVT risk related to in vitro fertilisation. By taking a proactive role in scientific popularisation and health literacy initiatives, nurses and midwives can help women better understand their risks, confidently participate in care planning, and make informed decisions that support safer pregnancy and postpartum outcomes.

## Author Contributions

M.X. and L.F. carried out the studies, participated in collecting data, and drafted the manuscript. Y.W. and T.Y. performed the statistical analysis and participated in its design. M.X. and L.F. participated in the acquisition, analysis, or interpretation of data and drafted the manuscript. All authors read and approved the final manuscript.

## Ethics Statement

This work has been carried out in accordance with the Declaration of Helsinki (2000) of the World Medical Association. This study was approved by the Medical Ethics Committee of the Second Affiliated Hospital of Xiamen Medical College [2023023]. All participants were informed about the study protocol and provided written informed consent to participate in the study.

## Consent

The authors have nothing to report.

## Conflicts of Interest

The authors declare no conflicts of interest.

## Supporting information


**Data S1:** nop270351‐sup‐0001‐Supinfo1.docx.

**Data S2:** nop270351‐sup‐0002‐Supinfo2.docx.

## Data Availability

The data that supports the findings of this study are available in the Supporting Information of this article.
