# Supplementary material for: The Knowledge, Attitude, and Practice Regarding Deep Vein Thrombosis Among Pregnant and Postpartum Women: The Mediating Effect of Attitude
Source: Nurs Open. 2025 Nov 14;12(11):e70351. doi: 10.1002/nop2.70351 (PMC12616879; doi:10.1002/nop2.70351)
Supplement: Supplementary file 1 — Data S1: nop270351‐sup‐0001‐Supinfo1.docx. [file NOP2-12-e70351-s001.docx]

| Dear participants：  We are the researchers of * hospital and sincerely invite you to participate in this study. This study aims to understand the knowledge, attitude and practice towards deep venous thrombosis among pregnant and postpartum women to provide a basis for the development of scientific intervention strategies, which may help more people improve their health in the future. Your participation in this study is voluntary, and if you agree to participate in this study, please refer to the instructions below.  1. Please complete the questionnaire. The answer has nothing to do with right or wrong and you only need to fill in according to the actual situation. After completion, please submit it in time.  2. This survey is a simple questionnaire, which cannot cause harm to your physical and psychological condition. However, this questionnaire will involve some privacy issues, such as gender, age, etc. Thus, we will keep the information strictly confidential and will not disclose any information that you fill in.  3. As a participant, you can always be informed about the progress of this study. If you decide to withdraw from the study, please let us know and your data will not be included in the results of the study.  Finally, thank you for taking the time out of your busy schedule to support our work!  □I acknowledge and agree to the use of the data collected for scientific research.  Scientific research：  Date: 202x –xx-xx |
| --- |

| **Part I-** **General information** | |
| --- | --- |
| **1.Your age：** | years old |
| **2.** **Are pregnant for the first time?** | a. Ture  b. False |
| **3.Your weight** | kg |
| **4.Your height** | cm |
| **5.** **Your place of residence：** | a. Country  b. Urban  c. Suburb |
| **6.** **Your ethnicity：** | a. Han  b. National minority  If you belong to minority, your nationality is: |
| **7.** **Your education level：** | a. Primary school and below  b. Junior High School/High School/Technical Secondary School  c. Junior college/Undergraduate  d. Master or above |
| **8.** **Your occupation：** | a. Regular employees  b. Part-time  c. Freelancing  d. Unemployed  e. Housewife/househusband  f. Students  h. Other: |
| **9. In the past year, your family's monthly per capita income (including income in kind and rental income, etc.): ______ yuan** | a.<2000  b.2000-5000  c.5000-10000  d.10000-20000  e.>20000 |
| **10. Type of medical insurance: (Multiple Choice)** | a. Medical insurance provided by the state (such as basic medical insurance for urban workers, new rural cooperative medical insurance, etc.)  b. Commercial medical insurance  c. None |
| **11. Do you suffer from any underlying diseases?** | a. Diabetes  B. High blood pressure  c. Kidney disease  d. Others:  e. None |
| **12.** **Whether you have a habit of smoking?** | a. Never  b. Smoked before, do not smoke now  c. Still smoking now |
| **13.** **Have you had any surgery performed on you?** | a. True  b. False |

**Part II-** **Knowledge**

**Please select "True" or "False" depending on your knowledge of the question. If you are unsure of the answer, select "Uncertain".**

| 1. Leg pain, swelling, discoloration are common symptoms of deep vein thrombosis | **a. True** | **b. False** | **c.** **Uncertain** |
| --- | --- | --- | --- |
| 2. Different thickness of lower limbs and fatigue after walking are early manifestations of deep vein thrombosis | **a. True** | **b. False** | **c.** **Uncertain** |
| 3. In-vitro fertilization increases the risk of deep vein thrombosis | **a. True** | **b. False** | **c.** **Uncertain** |
| 4. Factors such as advanced age, obesity, surgical history, long-term bed rest, and smoking will increase the risk of deep vein thrombosis | **a. True** | **b. False** | **c.** **Uncertain** |
| 5. Severe vomiting during pregnancy increases the risk of deep vein thrombosis | **a. True** | **b. False** | **c.** **Uncertain** |
| 6. Hypercoagulable state and venous stasis during pregnancy will increase the risk of deep vein thrombosis | **a. True** | **b. False** | **c.** **Uncertain** |
| 7. Cesarean section may increase the risk of blood clots during the puerperal period | **a. True** | **b. False** | **c.** **Uncertain** |
| 8. Deep vein thrombosis can be prevented by physical means (such as IPC, elastic stockings, etc.). | **a. True** | **b. False** | **c.** **Uncertain** |
| 9. Deep vein thrombosis can be prevented by anticoagulant drugs such as warfarin during pregnancy | **a. True** | **b. False** | **c.** **Uncertain** |
| 10. Deep vein thrombosis can be prevented by walking and increasing exercise | **a. True** | **b. False** | **c.** **Uncertain** |
| 11. Low-molecular-weight heparin heparin can be used for the prevention of deep vein thrombosis during pregnancy | **a. True** | **b. False** | **c.** **Uncertain** |
| 12. The use of low-molecular-weight heparin during pregnancy will harm the fetus, which cannot be used for the prevention of deep vein thrombosis | **a. True** | **b. False** | **c.** **Uncertain** |

**Part-III Attitude**

**Please select one of "strongly agree" to "strongly disagree" depending on whether you agree with the description in the question.**

| **1. Deep vein thrombosis can be fatal and requires special attention** | **a. Strongly agree** | **b. Agree** | **c. Neutrality** | **d. Disagree** | **e. Strongly disagree** |
| --- | --- | --- | --- | --- | --- |
| **2. Deep vein thrombosis generally occurs in older people, and I don't need to worry when I am young** | **a. Strongly agree** | **b. Agree** | **c. Neutrality** | **d. Disagree** | **e. Strongly disagree** |
| **3. All obese women will suffer from deep vein thrombosis** | **a. Strongly agree** | **b. Agree** | **c. Neutrality** | **d. Disagree** | **e. Strongly disagree** |
| **4. People who have been bedridden for a long time will suffer from deep vein thrombosis, and I don't need to worry about it in particular** | **a. Strongly agree** | **b. Agree** | **c. Neutrality** | **d. Disagree** | **e. Strongly disagree** |
| **5. The incidence of deep vein thrombosis is very low, I don't need to worry** | **a. Strongly agree** | **b. Agree** | **c. Neutrality** | **d. Disagree** | **e. Strongly disagree** |
| **6. Pregnant women are a high-risk group of deep vein thrombosis and need special attention to prevention** | **a. Strongly agree** | **b. Agree** | **c. Neutrality** | **d. Disagree** | **e. Strongly disagree** |
| **7. For pregnant women physical means are enough to prevent deep vein thrombosis** | **a. Strongly agree** | **b. Agree** | **c. Neutrality** | **d. Disagree** | **e. Strongly disagree** |
| **8. I don't want to prevent blood clots with drugs because drugs have the potential to affect my child** | **a. Strongly agree** | **b. Agree** | **c. Neutrality** | **d. Disagree** | **e. Strongly disagree** |
| **9. During the period of childbirth I should rest in bed and move less** | **a. Strongly agree** | **b. Agree** | **c. Neutrality** | **d. Disagree** | **e. Strongly disagree** |
| **10. Adequate cooperation with medical staff can help prevent deep vein thrombosis** | **a. Strongly agree** | **b. Agree** | **c. Neutrality** | **d. Disagree** | **e. Strongly disagree** |

**Part IV- Practice**

**Please choose the one that best meets your condition.**

| **1. I drink a lot of water in daily life** | **a. Always** | **b.** **Usually** | **c.** **Sometimes** | **d.** **Occasionally** | **e. Never** |
| --- | --- | --- | --- | --- | --- |
| **2. I try to wear loose clothes in daily life** | **a. Always** | **b.** **Usually** | **c.** **Sometimes** | **d.** **Occasionally** | **e. Never** |
| **3. I try to avoid staying in bed for a long time in daily life** | **a. Always** | **b.** **Usually** | **c.** **Sometimes** | **d.** **Occasionally** | **e. Never** |
| **4. I try to avoid high-fat meals in daily life** | **a. Always** | **b.** **Usually** | **c.** **Sometimes** | **d.** **Occasionally** | **e. Never** |
| **To prevent deep vein thrombosis：** | | | | | |
| **5. I am willing to follow my doctor's instructions to take drugs** | **a. Strongly agree** | **b. Agree** | **c. Neutrality** | **d. Disagree** | **e. Strongly disagree** |
| **6. I am willing to accept physical interventions such as IPC, elastic stockings, etc** | **a. Strongly agree** | **b. Agree** | **c. Neutrality** | **d. Disagree** | **e. Strongly disagree** |
| **7. I am willing to increase exercising** | **a. Strongly agree** | **b. Agree** | **c. Neutrality** | **d. Disagree** | **e. Strongly disagree** |
